# Use of morselized bone allograft in revision hip arthroplasty for massive acetabular defect: A systematic review and meta‐analysis

**DOI:** 10.1002/jeo2.70091

**Published:** 2024-12-04

**Authors:** Pietro Cimatti, Nicolandrea Del Piccolo, Benedetta Dallari, Alessandro Mazzotta, Dante Dallari

**Affiliations:** ^1^ Reconstructive Orthopaedic Surgery Innovative Techniques—Musculoskeletal Tissue Bank IRCCS Istituto Ortopedico Rizzoli Bologna Italy; ^2^ Department of Surgical Sciences and Innovative Technologies University of Bologna—Alma Mater Studiorum Bologna Italy

**Keywords:** acetabular defect, hip, morselized allograft

## Abstract

**Purpose:**

Many treatment options are available for the revision of large acetabular defects. Debate continues as to which technique is most effective. This meta‐analysis aimed to determine the rates of failure of acetabular bone defects Paprosky type III or American Academy of Orthopaedic types III–IV treated with morselized allograft in association with cemented cup or cementless cup or reinforcement devices.

**Methods:**

The US National Library of Medicine (PubMed/MEDLINE), EMBASE and the Cochrane Database of Systematic Reviews were queried for publications from January 1980 to 1 April 2024 utilizing keywords pertinent to total hip arthroplasty (THA), acetabular impaction bone grafting and revision THA. The main outcome measure was the 9‐year implant failure rate.

**Results:**

Thirty‐nine articles were eligible for inclusion in the current study. We found 41 treatment approaches that we grouped into three different treatment options: 1 = morselized allograft and cemented cup (10 studies); 2 =morselized allograft and cementless cup (nine studies); 3 = morselized allograft and device (22 studies). The overall implant failure rate was 2.1% (95% confidence interval [CI], 1.6%–2.8%) at a mean of 9.2 years. There was no significant difference in failure rates between different treatment options (1.6% [95% CI, 0.9%–2.6%]) for morselized allograft and cemented cup; 2.1% (95% CI, 1.4%–3.2%) for morselized allograft and cementless cup; 2.5% (95% CI, 1.7%–3.7%) for morselized allograft and device) between the three different types of treatment (heterogeneity between groups *p* = 0.351).

It was determined that the number one cause of failure was aseptic loosening (80.5%), followed by infection (13.1%) and dislocation (6.4%). THA with reinforcement devices has a higher incidence of infection (3.6 vs. 0.7%, *p* = 0.001) and dislocation (1.4 vs. 0.6%, *p* = 0.010) than THA with a cemented cup.

**Conclusions:**

The use of morselized allograft in hip revision of large acetabular defects has low implant failure rates, independently of the associated type of implant. Reinforcement devices increase the risk of re‐revision for infection and dislocation.

**Level of Evidence:**

Level III.

**Abbreviations**
AAOS: American Academy of Orthopaedic Surgeon
IBG: impaction bone grafting
THA: total hip arthroplasty

## INTRODUCTION

In the setting of acetabular cup revision arthroplasty, massive acetabular bone loss continues to be a critical issue for healthcare professionals [5, 17, 22, 26]. Two widely used classification systems have provided detailed anatomical information for defect‐specific preoperative planning: the American Academy of Orthopaedic Surgeons (AAOS) system and the system of Paprosky [36]. In general, the larger the defect, the more challenging is the acetabular revision. The most appropriate strategy for treating these acetabular defects is still under scrutiny [9]. Below are some common approaches and considerations.

Reinforcement devices such as metal meshes in conjunction with impaction bone grafting (IBG) have been utilized to minimize acetabular bone defects in the medial and superolateral walls in order to convert combined cavitary and segmental defects into isolated cavitary defects that facilitate IBG reconstruction [2, 10, 14, 28]. Histologic findings demonstrate successful vascularization and incorporation by creeping substitution of the impacted cancellous bone chips [38].

In contrast, some authors have reported cup migration and bone graft resorption after IBG in revision 60 surgery [12, 25]. Paprosky's classification system for acetabular defects includes type III defects, which are further subdivided into IIIA and IIIB categories. These subtypes exhibit significant differences in their anatomical characteristics and treatment challenges. Type IIIA defects are characterized by a significant acetabular defect with displacement of more than 3 cm superiorly and laterally from the obturator line, with the superior acetabular rim not providing support for the implant. The anterior and posterior columns are preserved, and the spherical cup has up to 50% contact with the parent bone. In contrast, Type IIIB defects involve severe osteolysis of the medial wall, complete disruption of the anterior column, extensive involvement of the posterior column and proximalization and medialization of the hip center, resulting in a limited remaining bone bed.

The primary goal of revision surgery for these defects is to achieve stable fixation of the prosthetic component while restoring an appropriate centre of rotation of the hip. Although various options for acetabular revision are available, no definitive treatment protocol is established. Complex periacetabular bone defects remain challenging, with no gold standard treatment. Larger hemispherical cups often suffice for most acetabular defects, but extensive segmental or uncontained defects may require bone augmentation.

Cages and rings, which may have osseointegration properties, can bridge defects and are often used in conjunction with cemented cups [14, 15]. Bone grafting, particularly IBG, is employed to address complex periacetabular bone defects. IBG is preferred over bulk grafts due to its superior osseointegration potential. Morselized bone chips are used to cover osteolytic areas and are impacted before implanting the acetabular component to achieve stable fixation. Morselized allografts can fill irregular bone defects effectively.

No consensus exists on the optimal technique for treating Paprosky Type IIIA or IIIB acetabular defects [1, 27]. Studies on IBG and cemented sockets for acetabular reconstruction report varying outcomes. Van Egmond et al. observed a 95% survival rate for aseptic loosening at 8.8 years of follow‐up [40]. Garcia‐Cimbrelo et al. reported an 82%–84% survival rate for Paprosky grades IIIA and IIIB at 8 years [11]. Schreurs et al. noted an 84% survival rate at 15 years [31], while Slooff et al. reported an 88% survival rate for any reason [33]. Welten et al. documented a 79% survival rate at 15 years [42].

Morselized bone allografts combined with acetabular components are widely used, particularly in cases of significant bone defects. Morselized bone allografts are advantageous in restoring bone stock.

Cementless hemispherical or elliptical implants may fail when acetabular defects exceed 50%, especially if the posterior column and dome are compromised. Strahl et al.'s systematic review and meta‐analysis revealed no significant difference in success rates between allograft types and acetabular cup fixation methods. Morselized allografts demonstrated a 3% higher success rate compared to bulk allografts [35].

Both graft types were applied in various Paprosky acetabulum defects.

In this systematic review and meta‐analysis, we compare the outcomes of treating Paprosky Type III A and III B defects with morselized bone allografts. On this background, we conducted a meta‐analysis to assess the role of morselized allograft combined with cemented cup, cementless cup, or reinforcement devices (cage, ring, mesh) for the management of massive acetabular defects (Paprosky type III A and III B or AAOS type III‐IV).

The acetabular defect Paprosky type III makes up to 95% of all cases of THA revisions.

The main aim of the study was to assess the revision rate of morselized allograft with cemented cup, cementless cup or reinforcement device used for the management of severe acetabular defects.

Secondary objective of this study was to assess the number of aseptic loosening, the number of infections and the number of implants with dislocation by type of acetabular implant.

## METHODS

### Search criteria

This meta‐analysis was based on Preferred Reporting Items for Systematic Reviews and Meta‐Analyses guidelines [24]. This study did not address the clinical outcomes after acetabular revision with allograft because we attempted to answer only the study question. The US National Library of Medicine (PubMed/MEDLINE), EMBASE and the Cochrane Database of Systematic Reviews were queried for publications from January 1980 to 1 April 2024 using keywords regarding total hip arthroplasty (THA), namely: acetabular bone defects, morselized bone allograft and revision THA. Only abstracts that evaluated utility of morselized bone grafting for reconstruction of massive acetabular bone defects were reviewed. The specific search terms are shown in Table 1.

**Table 1:** Search strategy.

Database	Pubmed, Embase, Cochrane
**Date**	1 April 2024
**Strategy**	#1 AND #2 AND #3
**Limit**	Human
**#1**	hip OR hips OR “joint, hip” OR coxa* OR acetabul* OR “cotyloid”
**#2**	“graft*” OR “bone graft*” OR “bone allograft*” OR “morselized allograft”
**#3**	“Acetabul*, defect” OR osteolysis OR osteolytic OR “bone deficiency”

### Inclusion and exclusion criteria

Any study reporting information on acetabular revision with bone allograft for large acetabular bone defects was considered potentially relevant and selected for primary review. There were no limitations for time period, language and time to follow‐up. The level of evidence was classified according to the definition given by the Oxford Centre for Evidence‐Based Medicine.

All levels of evidence assigned by the authors were included. All prospective, randomized, controlled studies (levels I and II) and all prospective or retrospective studies with or without control groups (levels III and IV) were accepted to be included in our study if they reported the incidence of acetabular cup re‐revision. Because most of the included studies were case series, we used the Newcastle–Ottawa Quality Assessment Scale to assess the methodological quality of the papers (Table 2).

**Table 2:** Quality assessment of the studies using Newcastle–Ottawa Scale for cohort studies.

**N. study**	**First author**	**Represent ativeness of the exposed cohort**	**Selection of the nonexposed cohort**	**Ascertainment of exposure**	**Demonstration that the current outcome of interest was not present at start of study**	**Comparability of cohorts on the basis of the design or analysis**	**Assessment of outcome**	**Was follow‐up long enough for outcomes to occur**	**Adequacy of follow‐up of cohorts**	**Quality score**
1	B. W. Schreurs	*	*	*	*		*	*	*	7
2	E. Garcia‐Cimbrelo	*	*	*			*	*		5
3	S. El‐Kawy	*	*	*			*	*		5
4	E. Garcia‐Cimbrelo	*	*	*	*		*	*	*	7
5	M. A. Buttaro	*	*	*	*	*	*	*	*	8
6	E. H. Van Haaren	*	*	*	*		*	*	*	7
7	G. Ullmark	*	*	*	*	*	*	*	*	8
8	W. S. Borland	*	*	*	*		*	*	*	7
9	N. van Egmond	*	*	*	*		*	*	*	7
10	S. Mehendale	*	*	*	*	*	*	*	*	8
11	E. Garcìa‐Rey	*	*	*	*		*	*	*	7
12	I. Kostensalo	*	*	*		*	*	*	*	7
13	S. S. Leopold	*	*	*	*		*	*	*	7
14	B. W. Schreurs	*	*	*	*			*		5
15	E. Scott Paxton	*	*	*	*		*	*		6
16	K. Lingaraj	*	*	*	*		*	*	*	7
17	D. A. Dennis	*	*	*	*			*	*	6
18	C. Wedemeyer	*	*	*			*	*	*	6
19	G. Köster	*	*	*	*		*	*	*	7
20	A. Herrera	*	*	*	*		*	*	*	7
21	C. J. Sutherland	*	*	*	*		*	*	*	7
22	C. L. Peters	*		*	*		*	*	*	6
23	M. K. Zehntner	*	*	*	*	*	*	*	*	8
24	S. W. Wachtl	*	*	*			*	*	*	6
25	E. Winter	*	*	*	*		*	*		6
26	J. Gallo	*	*	*	*		*	*	*	7
27	D. J. Berry	*		*	*		*	*	*	6
28	L. Jones	*	*	*		*	*	*	*	7
29	Q. Xiao	*	*	*			*	*	*	6
30	M. A. Buttaro	*	*	*		*	*	*	*	7
31	J. Buckup	*	*	*	*	*	*	*	*	8
32	B. Fink	*	*	*	*	*	*	*	*	8
33	I. Ilyas	*	*	*			*	*		5
34	J. Rosson	*	*	*	*	*	*	*	*	8
35	A. Gerber	*	*	*	*		*	*		6
36	E. Garcìa‐Rey	*	*	*	*		*	*		6
37	C. Yang	*	*	*	*	*	*	*	*	8
38	E. Quarto	*	*	*	*	*	*	*	*	8
39	X. Zhang	*	*	*	*	*	*	*	*	8

The inclusion criteria were (Table 3): [1] clinical trials investigating the outcome of primary and/or revision THA using morselized bone allograft for the treatment of large acetabular bone defects (Paprosky type 3a‐3b or AAOS type 3‐4). The exclusion criteria were (Table 3): [1] case report studies with less than five patients; [2] biomechanical studies; [3] studies dealing with femoral reconstruction; [4] studies without radiological outcomes and [5] studies with results including mixed types of implants without separate analysis.

**Table 3:** Selection criteria.

Inclusion criteria	Exclusion criteria
Treatment of massive acetabular bone defects	Treatment of minor acetabular bone defects
Use of morselized bone allograft	Use of autograft or structural allograft
Radiological outcomes	Studies with <5 patients

### Data collection

Two authors independently conducted the search. All authors compiled a list of articles not excluded after application of the inclusion and exclusion criteria. Discrepancies between the authors were resolved by discussion. During initial review of the data, the following information was collected for each study: title, author, year published, study design, sample size, indication for surgery, type of bone allograft, classification of acetabular defects, duration of follow‐up, reoperation rate. The level of evidence in the included studies was determined using the Oxford Centre for Evidence‐Based Medicine‐Levels of Evidence. The methodological quality of each study and the different types of detected bias were assessed independently by each reviewer with the use of the Newcastle–Ottawa Quality Assessment Scale (Table 2).

Studies in which the AAOS system or the Paprosky system were used to objectively define the acetabular defect were included. AAOS types 3 and 4 and Paprosky types 3A and 3B were rated as large acetabular defects and were those included in the present study. Secondary outcome measures were the number of aseptic loosening, the number of infections, and the number of 113 implants with dislocation [8, 11, 12, 13, 16, 19, 20, 21, 25, 29, 31, 32, 39, 41].

### Study selection

**Figure 1:**
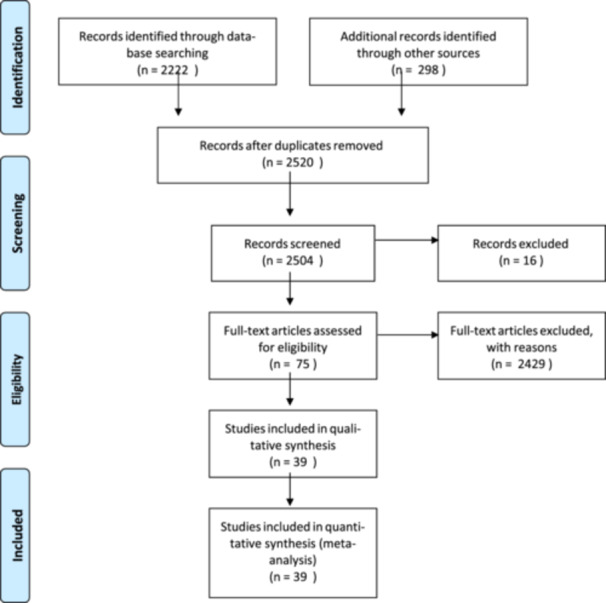
Prisma flow diagram.

In stage 1, we searched for all relevant articles electronically. We found a total of 2520 clinical studies on the revision of large acetabular defects with bone allograft. Following the removal of duplicate articles, the total number of studies used for this meta‐analysis was 2504. In stage 2, the abstracts of all the 2504 selected studies were checked manually in a primary screening by two independent reviewers (P. C. and A. M.). In stage 3, following application of these exclusion criteria, 75 articles were subject to a full‐text screening process by the two reviewers. Of these 75 articles, 36 were excluded from final analysis because they didn't deal with morselized allogenic bone graft. The eligible articles were reviewed for quality assessment using Newcastle–Ottawa Quality Assessment Scale and included in the systematic review and the meta‐analysis [34]. We did not consider a minimum level of quality to not exclude any study. The final analysis was therefore based on 39 articles (Figure 1). In stage 4, two reviewers checked the data independently in a standardized fashion.

The following information was collected for each study: title, author, year published, study design, number of patients, type of acetabular component fixation (cement vs. press‐fit vs. with reinforcement device), type of reinforcement device, type of allograft (morselized), classification of acetabular defects according to Paprosky and AAOS classifications, follow‐up length, radiographic loosening, revision and failure rates.

### Outcome measures

The primary outcome was the failure rate of the acetabular implant. The incidence of re‐revision was analysed by creating a forest plot. Furthermore, the incidence of re‐revision was analysed after pooling the number of revisions in each study.

A systematic review and meta‐analysis were conducted to evaluate the effectiveness of morselized bone.

Allografts in the treatment of Paprosky Type III A and III B acetabular defects. Outcomes were compared between the two defect types, including revision rates and the type of acetabular implants used.

Subgroup analysis was performed to determine if the type of surgery had a relationship with re‐revision of acetabular cup. These included the type of fixation (cemented vs. uncemented press‐fit vs. with reinforcement device), type of reinforcement device (cage, plate or mesh), type of allograft (morselized) and classification of the initial acetabular defect according to Paprosky and AAOS classifications. Secondary outcome measures were the number of aseptic loosening, the number of infection and the number of implant with dislocation.

### Statistical analysis

This analysis took study effects into account, and a random‐effects model was used for statistical analysis to calculate the risk ratio and 95% confidence interval (95% CI). The null hypothesis (the true effect size is 0) was rejected if the *p *< 0.05. To address the proportion of sampling error versus the true effect, we assessed the heterogeneity using Q statistics, and the degree of freedom to compute the *p* value that addresses the null hypothesis that the dispersion of the effect size was because of the random sampling error. We rejected the null hypothesis if *p *< 0.05, suggesting that the true effects varied. We also used the Q statistics to compute the I2, which shows that the proportion of dispersion in the effect sizes was because of true difference in the effect. If I2 equals 0, this suggests that all dispersion in the effect sizes can be attributed to the random sampling error. I2 describes the percentage of total variation across studies that is due to heterogeneity rather than chance. Negative values of I2 are put equal to 0 so that I2 lies between 0% and 100%. A value of 0% indicates no observed heterogeneity, and larger values show increasing heterogeneity. Following rule of thumb, we considered I2 >40% as substantial heterogeneity.

## RESULTS

**Figure 2:**
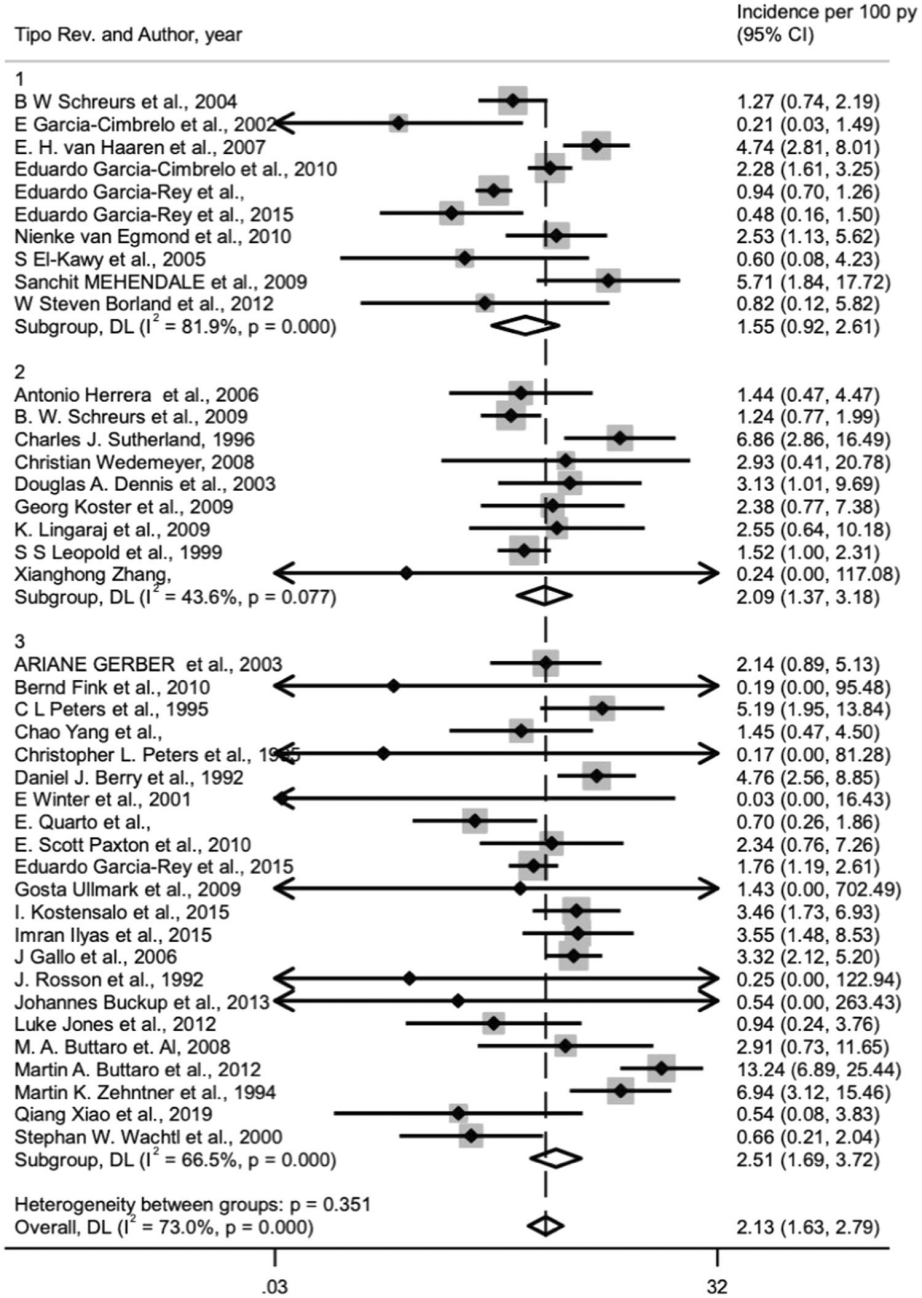
Forest plot for incidence of prosthesis failure in acetabular cemented cup [1], cementless cup [2] and antipro‐trusio devices [3] (1 vs. 2 *p* = 0.385, 1 vs. 3 *p* = 0.158, 2 vs. 3 *p* = 0.554).

Based on the random‐effects model for 39 studies, for a total of 1946 patients, treated with morselized allograft associated with different surgical procedures (cemented cup, cementless cup and antiprotrusio devices), the general incidence of prosthesis failure was 2.1% (95% CI, 1.6%–2.8%) (Figure 2).

### Cemented cup with allogenic morselized bone graft

Morselized bone allograft with cemented cup was the method of treatment used in 10 of the selected studies including a total of 891 patients. The incidence of prosthesis failure was 1.6% 180 (95% CI, 0.9%–2.6%) (Figure 2).

### Cementless cup with allogenic morselized bone graft

We included a total of nine studies that used a combination of morselized graft and cementless cup for a total of 326 patients. In this case, the failure rate was 2.1% (95% CI, 1.4%–3.4%) (Figure 2).

### Antiprotrusio devices with allogenic morselized bone graft

**Figure 3:**
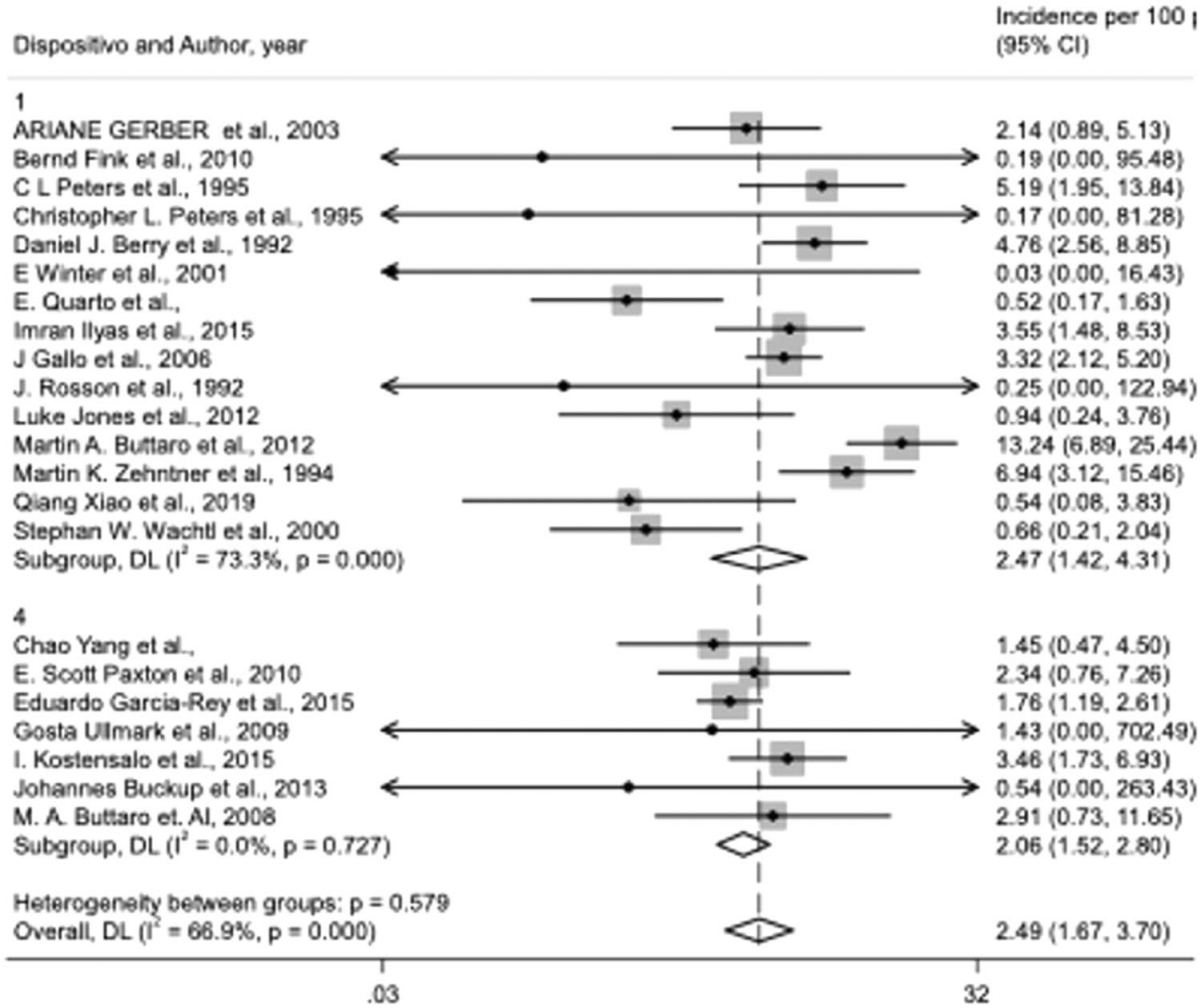
Forest plot for prosthesis failure rate in acetabular cup reinforced with cage [1] or mesh [4].

Morselized bone allograft with antiprotrusio devices (cage, plate or mesh) was the method used in 22 of the selected studies (729 patients). Based on the random‐effects model for 22 studies with morselized bone allograft, the incidence of prosthesis failure was 2.5% (95% CI, 1.7%–3.7%) (Figure 2). Subgroup analysis comparing the failure rates among cage, plate or mesh in association with morselized allograft, showed no significant differences (*p* = 0.579) with the lower failure rate (2.1%) with mesh and the worse with cage (2.5%) (Figure 3).

### Comparison among different surgical procedures

Comparing these failure rates, using different type of devices associated to morselized bone graft there was no significant difference (*p* = 0.351) between the different types of intervention with the use of morselized bone allograft in Paprosky 3 and AAOS 3 and 4 acetabular defects (Figure 2).

### Comparison of Paprosky Type 3A and 3B acetabular defects Treated with morselized bone allograft

In the present study, we analysed data from nine studies involving Paprosky Type 3A acetabular defects, which included a total of 343 patients, and 10 studies involving Paprosky Type 3B acetabular defects, with a total of 332 patients. The re‐revision rate for Paprosky Type 3A defects was 1.45% (95% CI, 0.78%–2.70%), while for Paprosky Type 3B defects, it was 1.65% (95% CI, 1.11%–2.46%).

**Figure 4:**
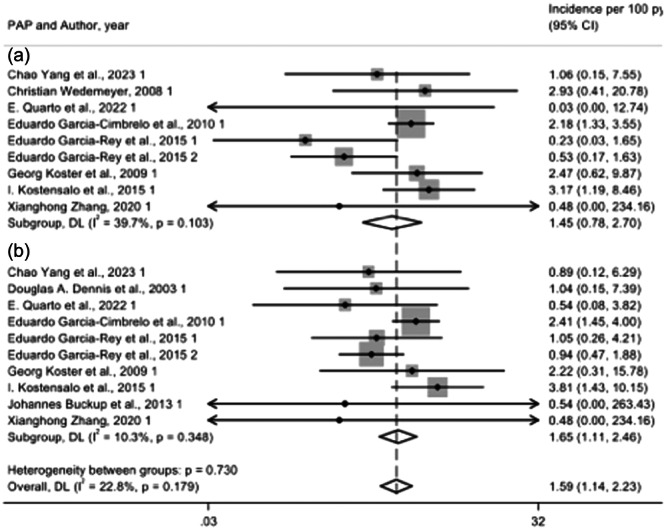
Re‐revision incidence: Paprosky 3a versus Paprosky 3b.

There was no significant difference (*p *= 0.730) in the re‐revision rates between the two types of Paprosky acetabular defects (Figure 4).

When comparing revision rates associated with different types of acetabular implants (cemented cups, cementless cups, reinforcement devices) in conjunction with morselized bone allograft, no significant differences were observed between the interventions for Paprosky Type 3A and 3 B defects. Specifically, the use of cemented cups resulted in a re‐revision incidence of 0.88% (95% CI, 0.10%–7.55%) for Paprosky Type 3A defects and 2.07% (95% CI, 1.10%–3.88%) for Paprosky Type 3 B defects (*p* = 0.454). For cementless cups, the re‐revision incidence was 2.47% (95% CI, 0.81%–7.53%) for Paprosky Type 3A and 1.44% (95% CI, 0.37%–5.57%) for Paprosky Type 3B (*p* = 0.545). The use of reinforcement devices resulted in a re‐revision incidence of 1.27% (95% CI, 0.36%–4.43%) for Paprosky Type 3A defects and 1.53% (95% CI, 0.60%–3.86%) for Paprosky Type 3B defects (*p* = 0.817).

### Leading cause of prosthesis failure

**Figure 5:**
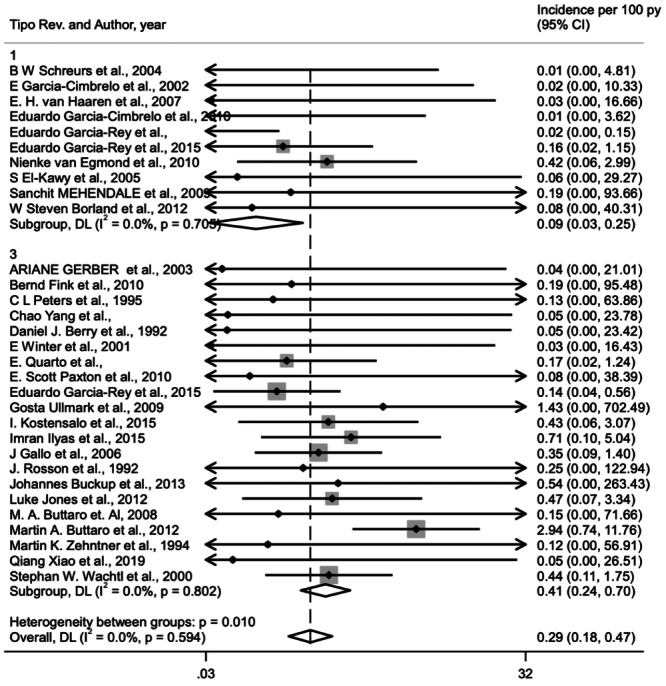
Infection incidence: cemented cup versus reinforcement device.

**Figure 6:**
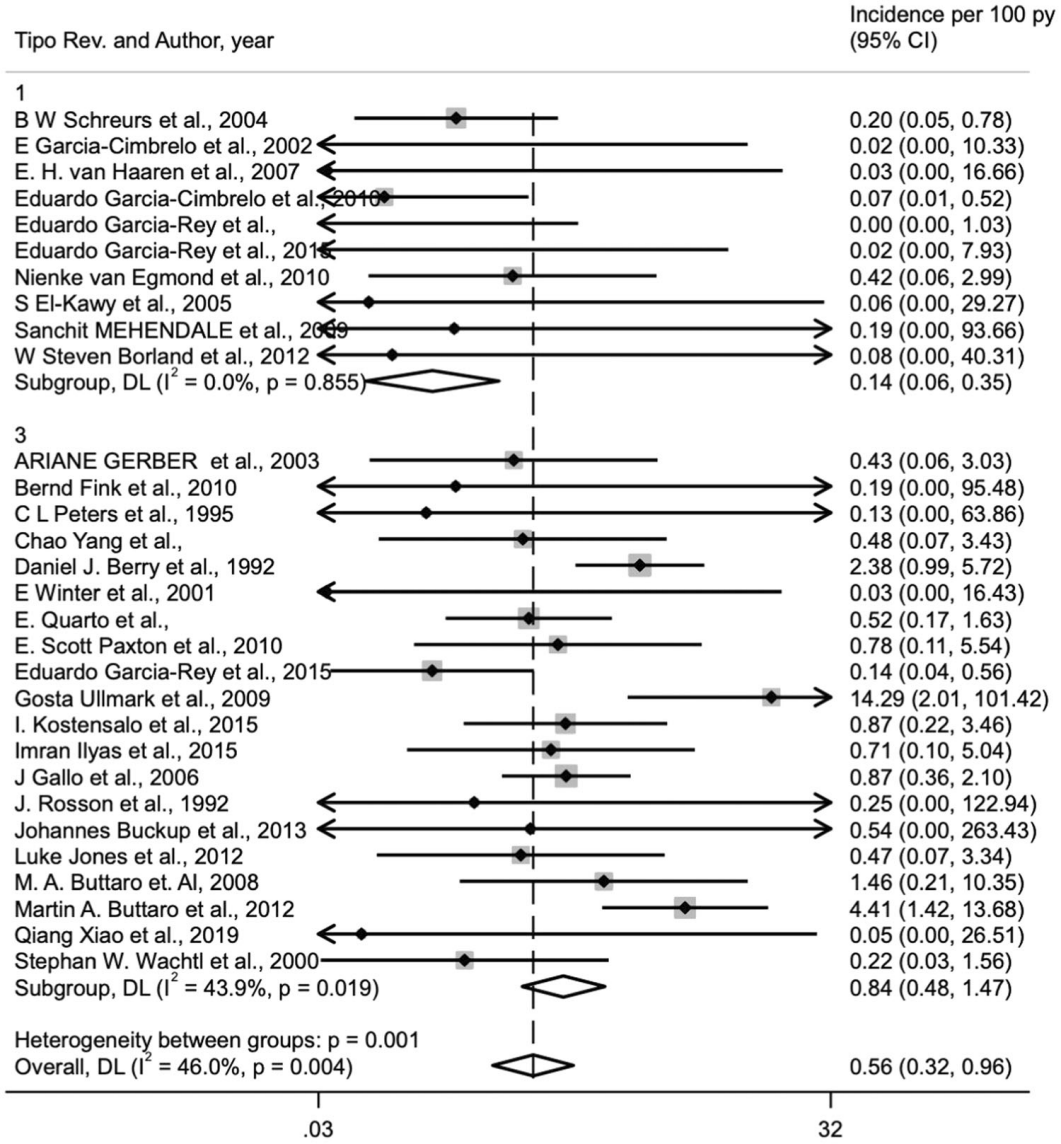
Dislocation incidence: cemented cup versus reinforcement device.

Of the failures, aseptic loosening accounted for the largest proportion of the total number of failures (227/282—80.5%). This was followed by infection (37/282—‐13.1%) and dislocation (18/282—2256.4%) (Table 4). The data from the 39 studies was further broken down into patient populations based on three different surgical treatment options (Table 4). 1946 revision hips were reviewed and studied to investigate outcomes given types of surgical treatment and failure classification. There were 891 revisions using cemented cup, 326 revisions using cementless cup and 729 revisions using reinforcement devices. The total number of failures were 118, 56 and 109, respectively. Individuals receiving a revision THA with reinforcement devices are at a higher risk of infection (*p *= 0.001) and dislocation (*p *= 0.010) than patients treated with THA and cemented cup (Table 5, Figures 5 and 6). Mode of failure that showed no difference between these groups was aseptic loosening. Surgical treatment with cementless cup showed no significant difference from cemented cup and reinforcement devices in terms of incidence of aseptic loosening, dislocation and infection (Table 5).

**Table 4:** Breakdown of prosthesis failure in different surgical procedures.

Failure mode	Cemented cup	Cementless cup	Reinforcement devices	Overall
N. Patients	891	326	729	1946
Total failures	118 (13.24%)	56 (17.18%)	109 (14.95%)	283 (14.54%)
Aseptic loosening	62 (6.96%)	48 (14.72%)	72 (9.88%)	182 (9.35%)
Infection	6 (0.67%)	5 (1.53%)	26 (3.57%)	37 (1.9%)
Dislocation	5 (0.56%)	3 (0.92%)	10 (1.37%)	18 (0.92%)

**Table 5:** Prosthesis failure in different surgical procedures.

Failure mode	Cemented cup	Cementless cup	Reinforcement devices	*p* Value cemented cup vs cementless cup	*p* Value cemented cup vs reinforcement devices	*p* Value cementless cup vs reinforcement devices
N. patients	891	326	729			
Total failures						
N	118	56	109			
Incidence *per* 100 py (95%CI)	1.55 (0.92, 2.61)	2.09 (1.37, 3.18)	2.51 (1.69, 3.72)	0.385	0.148	0.530
Dislocation	3	3	12			
N						
Incidence *per* 100 py (95%CI)	0.09 (0.03, 0.25)	0.17 (0.06, 0.47)	0.41 (0.24, 0.70)	0.367	0.010	0.140
Infection	4	5	28			
N						
Incidence *per* 100 py (95%CI)	0.14 (0.06, 0.35)	0.30 (0.11, 0.85)	0.84 (0.48, 1.47)	0.282	0.001	0.087
Aseptic loosening	111	48	64			
N						
Incidence *per* 100 py (95%CI)	1.40 (0.81, 2.43)	1.88 (1.14, 3.09)	1.95 (1.46, 2.60)	0.441	0.300	0.900

## DISCUSSION

Bone stock restoration is a critical aspect of acetabular revision surgery, and it plays a pivotal role in the success and longevity of the implant and the overall well‐being of the patient. Morselized allografts to restore bone stock for massive acetabular bone defect in THA can be a valuable tool in reconstructing bone deficiencies, but there are concerns about their long‐term stability and potential complications, including allograft resorption. Morselized grafts can be used to fill defects of any shape, thus providing optimal adaptation to the host bone and implant. Over time, morselized allografts can integrate with the patient's own bone as they undergo a process of incorporation and remodelling. It has been reported that morselized allografts are superior in terms of revascularization compared with other type of allograft‐like bulk grafts [15, 18]. In a histologic study, Ullmark and Obrant suggested that IBG combined with a cemented polyethylene liner resulted in an early formation of fibrous tissue and patches of new bone. According to that study, the healing process of IBG was similar to fracture healing with only one exception. Specifically, the endochondral bone formation may take place in a bone graft bed consisting of morselized and impacted allograft that contains a fibrin clot [38].

Recent studies have demonstrated favourable outcomes with morselized allografts and augmentation devices. For example, Garcìa Rey et al. used meshes for uncontained defects and morselized allograft chips, observing durable results with large defects [12]. Borland et al. employed Trabecular Metal augments followed by morselized bone grafting and cemented sockets, achieving satisfactory clinical and radiographic results [3].

Sloof et al. proposed using flexible meshes to convert uncontained defects into contained ones, followed by impaction of morselized allografts and cement fixation [33]. Yang et al. found morselized bone allograft and metal mesh with a cemented acetabular cup effective for severe acetabular defects [43]. Buckup et al. highlighted the utility of morselized bone allograft combined with mesh and cemented cups for Paprosky III B defects, reporting no re‐revisions or radiographic failures [4]. Impaction grafting with reconstruction meshes allows for stable cavity creation and accurate cup placement. Buttaro et al. noted that while impaction grafting with reconstruction meshes and cemented acetabular components is a reliable solution for medium‐severity defects, it may not be suitable for severe combined deficiencies [6].

Furthermore, concerns persist regarding the use of morselized allografts, particularly with respect to their long‐term stability and potential complications, such as allograft resorption and risk of infection. In the current study, we demonstrated that the implant survival rates are extremely high at a mean follow‐ up of 9.2 years. Cemented cups, uncemented cups and antiprotrusio devices showed success rates of 98.45%, 97.91% and 97.51%, respectively. Massive acetabular defects, therefore, can be treated with optimal success rate by using the morselized graft that is the associated device. The findings suggest that morselized bone allografts are effective in treating both Paprosky Type III A and III B acetabular defects, with no significant differences in outcomes between the two defect types. The choice of acetabular implant and reinforcing device does not appear to affect the success rate significantly. Aseptic loosening is one of the major causes of re‐revision in hip arthroplasty, accounting for a significant percentage of total failures. Accordingly, this study found that aseptic loosening was the major cause of re‐revision accounting for 80.5% of the total failures. We also investigated the relative risks for re‐revision associated with the use of different surgical procedures, specifically the use of cemented cups, cementless cups and reinforcement devices. Our finding that the use of reinforcement devices increases the risk of re‐revision for infection and dislocation aligns with some clinical considerations. Reinforcement devices, by design, can provide additional surfaces where bacteria may potentially colonize, increasing the risk of postoperative infection. In addition, reinforcement devices require wider surgical exposure and long operating times. These two factors further increase the risk of infection. There may be changes in hip biomechanics that could affect the risk of dislocation.

## LIMITATIONS

The main limitation of this systematic review and meta‐analysis is that the source studies did not provide high‐quality evidence. In view of the fact that, contrary to Paprosky's original recommendation, morselized bone allografts were also used for type IIIA and IIIB acetabular defects [27]. The differences in surgical approach, postoperative care protocols, patient demographics, primary disease, number of cases and time to follow‐up created limited comparability. Furthermore, metanalytical results were affected by publication bias. However, for the outcome parameters examined, only follow‐up studies were suitable and applicable.

## CONCLUSION

The use of morselized bone allografts in the treatment of acetabular revision, including both cementless and cemented techniques, can be an effective approach for addressing massive bone defects, specifically those classified as AAOS Type 3‐4 or Paprosky Type 3A–3B.

Morselized bone allografts, combined with various acetabular components and reinforcement techniques, provide a reliable option for managing complex Paprosky acetabular defects. Further research, including randomized controlled trials, is needed to refine treatment protocols and improve outcomes for severe acetabular defects. It would be interesting to conduct randomized controlled trials to compare massive allografts with clamped allografts and to refine treatment protocols for severe acetabular defects. Reinforcement device increases the risk of re‐revision for infection and dislocation.

The poorer outcomes related to infections associated with the use of reinforcement devices are likely due to increased surgical aggressiveness and prolonged operating times. Ensuring proper implant positioning and soft tissue tension during surgery is essential to minimize dislocation risk, but the complexity of revision cases can make achieving optimal stability more challenging [37].

Mitigating the risk of infection involves a comprehensive approach that includes consideration of patient‐specific factors such as patient's age, overall health, immune status and activity level [7, 23, 30]. Patients should be engaged in thorough discussions with their surgical teams to understand the rationale behind the chosen surgical approach and the associated risks and benefits.

## AUTHOR CONTRIBUTIONS

The author(s) read and approved the final manuscript.

## CONFLICT OF INTEREST STATEMENT

The authors declare no conflicts of interest.

## ETHICS STATEMENT

The authors have nothing to report.

## Data Availability

The data sets used and analysed during the current study are available from the corresponding author on reasonable request.
